# Bioenergy therapies as a complementary treatment: a systematic review to evaluate the efficacy of bioenergy therapies in relieving treatment toxicities in patients with cancer

**DOI:** 10.1007/s00432-022-04362-x

**Published:** 2022-09-27

**Authors:** M. Hauptmann, S. Kutschan, J. Hübner, J. Dörfler

**Affiliations:** Klinik für Innere Medizin II, Hämatologie und Internistische Onkologie, Universitätsklinikum Jena, Am Klinikum 1, 07747 Jena, Germany

**Keywords:** Complementary and alternative medicine, Bioenergy therapies, Reiki, Therapeutic Touch, Healing Touch, Polarity Therapy

## Abstract

**Purpose:**

Bioenergy therapies are among the popular alternative treatment options for many diseases, including cancer. Many studies deal with the advantages and disadvantages of bioenergy therapies as an addition to established treatments such as chemotherapy, surgery, and radiation in the treatment of cancer. However, a systematic overview of this evidence is thus far lacking. For this reason, the available evidence should be reviewed and critically examined to determine what benefits the treatments have for patients.

**Methods:**

In June 2022, a systematic search was conducted searching five electronic databases (Embase, Cochrane, PsychInfo, CINAHL and Medline) to find studies concerning the use, effectiveness and potential harm of bioenergy therapies including Reiki, Therapeutic Touch, Healing Touch and Polarity Therapy on cancer patients.

**Results:**

From all 2477 search results, 21 publications with 1375 patients were included in this systematic review. The patients treated with bioenergy therapies were mainly diagnosed with breast cancer. The main outcomes measured were anxiety, depression, mood, fatigue, quality of life (QoL), comfort, well-being, neurotoxicity, pain, and nausea. The studies were predominantly of moderate quality and for the most part found no effect. In terms of QoL, pain and nausea, there were improved short-term effects of the interventions, but no long-term differences were detectable. The risk of side effects from bioenergy therapies appears to be relatively small.

**Conclusion:**

Considering the methodical limitations of the included studies, studies with high study quality could not find any difference between bioenergy therapies and active (placebo, massage, RRT, yoga, meditation, relaxation training, companionship, friendly visit) and passive control groups (usual care, resting, education). Only studies with a low study quality were able to show significant effects.

**Supplementary Information:**

The online version contains supplementary material available at 10.1007/s00432-022-04362-x.

## Introduction

Complementary and alternative medicine such as bioenergy therapies are becoming increasingly popular in Europe. The 1-year prevalence among patients in the UK is 41.1% and the lifetime prevalence is 51.8%, meaning that one in two patients will come into contact with these treatments during their lifetime (Posadzki et al. 2013). Bioenergy therapies include Reiki as well as Therapeutic Touch, Healing Touch and Polarity Therapy.

Reiki is a form of energy healing, originating in Japan, in which it is assumed that appropriately trained practitioners create a channel for the Reiki energy, usually by placing their hands on patients (Reiki-Verband-Deutschland 2022). However, some practitioners prefer instead to hold the hands a few inches above the body of the person being treated. It is assumed that the therapist is not working with his own energy. Therefore, Reiki therapists can also treat themselves. In theory, even animals and plants can be treated.

Therapeutic Touch was developed at New York University as a method of laying on hands, in which the practitioner does not touch the patient (European Therapeutic Touch Institute 2007). The principle is based on the assumption that the therapist can use his hands to feel and harmonize the human energy field, which is formed in the body and extends beyond the body’s boundaries. It is assumed that this enables a conscious direction of the energy.

Healing Touch is based on the belief of human energy fields (Healing Touch Deutschland 2019). The therapist therefore tries to establish a relationship with the patient’s energy centers by using gentle touches on the hands. This is intended to stabilize the patient’s energy and make it stronger.

Polarity Therapy is based on the principle of polarities, i.e., energies that move between two opposite poles (American Polarity Therapy Association 2022). The treatment aims to influence the life energy through the laying on of hands in such a way that blockages are released. The aim is to make energies flow freely so that diseases or problems are solved.

This review aims to examine clinical studies on the effects of bioenergy therapies on anxiety, mood, depression, fatigue, QoL, comfort, well-being, neurotoxicity, pain and nausea, and on the side effects of cancer treatments or symptoms due to cancer. In addition, an investigation of any side effects associated with bioenergy therapies will be undertaken.

## Methods

### Study selection

A systematic search was conducted using five databases [Medline (Ovid), CINAHL (EBSCO), EMBASE (Ovid), Cochrane CENTRAL and PsycINFO (EBSCO)] in June 2022. For each of these databases a complex search strategy was developed, consisting of a combination of MeshTerms, keywords and text words in different spellings connected to cancer and bioenergy therapies (see search string, supplementary material). After importing the search results into EndNote X6, all duplicates were removed and a title- abstract- screening was carried out by two independent reviewers (MH and JD). In case of disagreement, consensus was achieved through discussion. When title and abstract did not have sufficient information for screening purposes, a full-text copy was retrieved as well. After that, all full texts were retrieved and screened again independently by both reviewers. Finally, 21 publications were analyzed in this review, consisting of 21 RCTs.

### Eligibility criteria

We included systematic reviews and randomized controlled trials (RCTs) if they reported patient-relevant outcomes (anxiety, mood, depression and fatigue) in treating adult cancer patients with Reiki, Therapeutic Touch, Healing Touch and Polarity Therapy as interventions. Type of treatment, frequency and duration were extracted. Because of the wide range of application fields, all cancer entities were included. Included patients were characterized by type and stage of cancer, type of treatment (e.g., chemo-, radiotherapy, operation), age and gender. Any kind of comparison was eligible in this review. This included watch and wait, standard care, sham and placebo. Criteria for rejecting studies were primary prevention, grey literature, other publication type than primary investigation/report (e.g., comments, letters, abstracts), studies with children (under the age of 18) or precancerous conditions, preclinical studies and other study types (non-randomized controlled studies, one-armed/non-controlled studies, case report or series, cohort/case–control studies). Additionally, studies were excluded if they reported no patient centered outcomes (laboratory parameters). Language restrictions were made to English and German. A recently published, high-quality review on this topic included a 1998 publication as the oldest publication, using a search string without time window (Agdal et al. 2011). Therefore, we limited our search to the year 1995.

### Study methodological quality appraisal

Data extraction was performed by one reviewer (MH) and controlled by two independent reviewers (JD, JH). As a template for data extraction, the evidence tables from the national Guideline on Complementary and Alternative Medicine in Oncological Patients of the German Guideline Program in Oncology (Deutsche Krebsgesellschaft 2019) were used. The risk of bias in the included studies was analyzed with the SIGN- Checklist for controlled trials Version 2.0 and AMSTAR-2 instrument for systematic reviews or meta-analyses (Shea 2017). The included studies were rated using the Oxford criteria. Additional criteria concerning methodology were size of population, application of power analysis, dealing with missing data and drop-out (report of drop-out reasons, application of intention-to-treat-analysis), adequacy of statistical tests (e.g., control of premises or multiple testing) and selective outcome reporting (report of all assessed outcomes with specification of statistical data as the *p* value). In addition, blinding of researchers, blinding of outcome assessment and comparability of groups before treatment, not only in terms of demographic variables but also concerning the baseline values, were examined.

## Results

### Results of the search

**Fig. 1:**
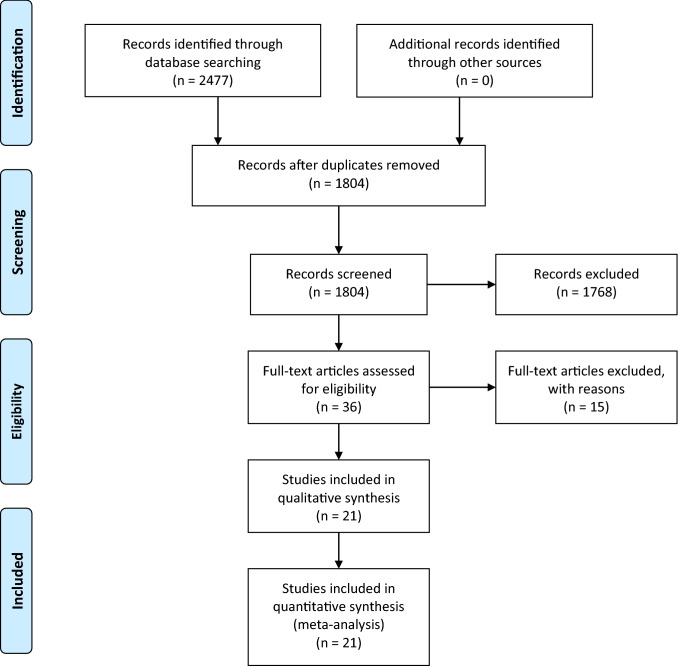
PRISMA diagram

The systematic research revealed 2477 results. No studies were added by hand search. At first, duplicates were removed, leaving 1804 studies. After screening title and abstract, 36 studies remained for complete review. Finally, 21 publications were analyzed in this review, consisting of 21 RCTs (see PRISMA diagram, Fig. 1). A detailed characterization of the studies included may be seen in Table 1 (see supplementary material).

### Description of interventions studies

Concerning all relevant studies, 1375 patients were included. The mean age of patients was 46.66 years, with a range of ages from 18 to 91 years. 884 participants were female (80.15%) and 219 male (19.85%). Two publications did not provide information on gender distribution (Olson et al. 2003; Catlin and Taylor-Ford 2011). The main outcomes measured were anxiety, mood, depression, fatigue, QoL, comfort, well-being, neurotoxicity, pain, and nausea. Eight RCTs examined Reiki (Alarcao and Fonseca 2016; Beard et al. 2011; Catlin and Taylor-Ford 2011; Clark et al. 2012; Olson et al. 2003; Orsak et al. 2015; Potter 2007; Tsang et al. 2007), eight RCTs examined Therapeutic Touch (Aghabati et al. 2010; Frank et al. 2007; Giasson and Bouchard 1998; Matourypour et al. 2015, 2016; Samarel et al. 1998; Tabatabaee et al. 2016; Vanaki et al. 2016), three RCTs examined Healing Touch (FitzHenry et al. 2014; Lutgendorf et al. 2010; Post-White et al. 2003) and two RCTs examined Polarity Therapy (Mustian et al. 2011; Roscoe et al. 2005). As active control groups, a placebo group was used in nine studies (Aghabati et al. 2010; Alarcao and Fonseca 2016; Catlin and Taylor-Ford 2011; FitzHenry et al. 2014; Frank et al. 2007; Matourypour et al. 2015, 2016; Vanaki et al. 2016; Tabatabaee et al. 2016), massage in two studies (Mustian et al. 2011; Post-White et al. 2003) and in one study each, relaxation response therapy (RRT) (Beard et al. 2011), yoga (Clark et al. 2012), meditation (Clark et al. 2012), relaxation training (Lutgendorf et al. 2010) as well as companionship (Orsak et al. 2015). Thirteen studies had a passive control group consisting of usual care (Aghabati et al. 2010; Beard et al. 2011; Catlin and Taylor-Ford 2011; Lutgendorf et al. 2010; Matourypour et al. 2015, 2016; Mustian et al. 2011; Orsak et al. 2015; Post-White et al. 2003; Potter 2007; Roscoe et al. 2005; Tabatabaee et al. 2016; Vanaki et al. 2016), four studies had a rest group as passive control group (Giasson and Bouchard 1998; Olson et al. 2003; Samarel et al. 1998; Tsang et al. 2007) and one study had education (Clark et al. 2012). The included cancer types were predominantly breast cancer but hematological cancer, lymphoma, prostate cancer, cervical cancer as well as colorectal cancer were also investigated. Detailed characterization of the included studies may be seen in Table 1 (see supplementary material).

### Excluded studies

Eleven systematic reviews were excluded due to insufficient relevance considering bioenergy therapies or cancer patients and due to missing risk of bias information or the included RCTs being already examined individually. The four excluded RCTs did not relate to Reiki, Therapeutic Touch, Healing Touch or Polarity Therapy or the full text was not available or no relevant endpoint was assessed. A list of excluded studies can be seen in Table 2 (see supplementary material).

#### Risk of bias of included studies

The results of the risk of bias assessment are presented in Tables 3 and 4 (see supplementary material). Three of the included studies have high quality (FitzHenry et al. 2014; Lutgendorf et al. 2010; Post-White et al. 2003), fifteen have moderate quality (Aghabati et al. 2010; Alarcao and Fonseca 2016; Beard et al. 2011; Catlin and Taylor-Ford 2011; Clark et al. 2012; Frank et al. 2007; Giasson and Bouchard 1998; Mustian et al. 2011; Olson et al. 2003; Orsak et al. 2015; Potter 2007; Roscoe et al. 2005; Samarel et al. 1998; Tabatabaee et al. 2016; Tsang et al. 2007) and three poor quality (Matourypour et al. 2015, 2016; Vanaki et al. 2016).

#### Effect of interventions

##### Reiki

Eight RCTs included Reiki as intervention (Alarcao and Fonseca 2016; Beard et al. 2011; Catlin and Taylor-Ford 2011; Clark et al. 2012; Olson et al. 2003; Orsak et al. 2015; Potter 2007; Tsang et al. 2007). The primary outcomes were anxiety, depression, mental stress, symptom distress, mood disturbances, mindfulness, fatigue, QoL, comfort, well-being, neurotoxicity and pain.

*Anxiety and depression*: the effects of Reiki on anxiety and depression were examined in three RCTs (Beard et al. 2011; Potter 2007; Tsang et al. 2007). Prostate cancer patients (*n* = 54) who underwent external radiation therapy were examined over 12 weeks after radiation and showed no significant differences between Reiki, relaxation response therapy (RRT) with cognitive restructuring and wait-list for anxiety and depression (Beard et al. 2011). It should be noted that the Reiki group received an intervention twice a week, whereas the RRT group only received an intervention once a week. In a sample of patients examined one week before and one week after a breast biopsy (*n* = 35), no significant differences were found between Reiki and conventional care with regard to anxiety and depression (Potter 2007). Reiki was able to achieve a significant improvement in anxiety but was not superior to conventional care. The study is not representative overall, as the study population consisted largely of health care professionals. The study by Tsang et al. (2007) found no significant differences after the fifth application between the Reiki and rest group regarding anxiety.

Regarding the effect of Reiki on anxiety, none of the studies provides evidence that Reiki is superior to the other active and passive treatments. Considering the effects on depression, only one study found a significant improvement in patients who were classified as depressed at the start of the study (Beard et al. 2011). However, the same significant results were detectable in the RRT and wait-list group. When looking at patients classified as suffering from anxiety at the start of the study, a significant improvement was only found in the RRT group, but not in the Reiki group.

*Mental stress, symptom distress, mood disturbances and mindfulness*: the effects of Reiki on mental stress, symptom distress, mood disturbances and mindfulness were examined in two RCTs (Clark et al. 2012; Orsak et al. 2015). The study by Clark et al. (2012) examined cancer patients with polyneuropathy (*n* = 36) over 6 weeks and found no significant effect with regard to mindfulness and mental stress with the subscales somatization, depression and anxiety compared to education, yoga and meditation. At the beginning of the study the number of participants was already low, and during the study, more participants dropped out. Furthermore, the patients had to self-report their hospital records, which may have led to possible misreporting. The study by Orsak et al. (2015) examined a sample of breast cancer patients (*n* = 36) over four cycles of chemotherapy and found no significant differences between the groups (Reiki, companion, usual care). In contrast, significant differences in the groups over time (*p* < 0.001) were found for mood disturbances and the confusion subscale, with Reiki having better scores than usual care and worse than companion. The same results were found for the subscale mental vigor, whereby Reiki had the worst values. No significant differences were found in the subscales anxiety, depression, anger, and fatigue. For short-term effects on mood disturbances and components such as fear, depression, anger, confusion and mental vigor, no significant differences were found over time between the groups. For fatigue only, there was a significant difference between the groups over time (*p* < 0.001), with worsened scores in the usual care group and improved scores in the Reiki and companion group. It should be mentioned that the companion group had already reported better mood at the beginning of the study. In addition, due to the study design, blinding could not be achieved, which was also the case in the other studies that investigated Reiki.

Overall, neither study provides evidence for the benefits of Reiki beyond placebo in terms of improving mental stress.

*Fatigue*: a crossover study examined patients with different types of cancer (*n* = 16) (Tsang et al. 2007). Fatigue was recorded using two different questionnaires (Functional Assessment of Cancer Therapy Fatigue subscale and Edmonton Symptom Assessment System). In the first assessment, which was done after the seventh Reiki intervention (before crossover), there were no significant differences between the Reiki and resting group (*p* = 0.236). In the second assessment, after five applications of the respective treatment (before crossover), significantly better values were found in the Reiki group compared to the resting group (*p* < 0.01). After a subsequent wash-out phase of one week without treatment, no significant differences were found between the groups (*p* = 0.215). As the overall quality of the study was poor, the results are not reliable. In particular, attention and expectation effects due to the lack of a placebo group cannot be ruled out or may be regarded as likely. Only one of two questionnaires had significantly better values in the Reiki group compared to the group simply resting. However, these differences were only found after the 5th application in each case and were no longer detectable after a 1-week wash-out phase without treatment.

Overall, no long-term positive effects of Reiki could be found regarding fatigue.

*Quality of life, comfort and well-being*: the effects of Reiki on quality of life (QoL), comfort and well-being were examined in seven RCTs (Alarcao and Fonseca 2016; Beard et al. 2011; Catlin and Taylor-Ford 2011; Clark et al. 2012; Orsak et al. 2015; Tsang et al. 2007; Olson et al. 2003), of which four found positive effects compared with sham, rest and standard care (Alarcao and Fonseca 2016; Catlin and Taylor-Ford 2011; Tsang et al. 2007; Olson et al. 2003). A sample of hematological cancer patients (*n* = 116) was examined over 4 weeks. There were significant differences in favor of the Reiki arm compared to the sham arm with regard to the physical (*p* = 0.015), social (*p* = 0.0005) and the surrounding domain of QoL (*p* = 0.0075), as well as the score for each domain (*p* = 0.035) (Alarcao and Fonseca 2016). The study is of poor quality and the validity of the results must be questioned. A complete comparability of the groups cannot be assumed, and the allocation to the individual groups does not appear to be entirely random. Another study (*n* = 16) found similar results compared to rest period as intervention (*p* = 0.04) (Tsang et al. 2007). Due to the lack of a placebo group and the small number of participants, the results are not reliable. The study by Olson et al. (2003) examined a sample of patients with cancer pain (*n* = 24) and found a significant improvement regarding the psychological component of QoL in the Reiki group compared to rest time after 7 days (*p* = 0.002). In contrast, a sample of prostate cancer patients (*n* = 54) showed no significant difference between Reiki, relaxation response therapy (RRT) and wait-list in total scores for QoL. Only on one of the four QoL subscales (emotional well-being), a significant difference was found in favor of RRT compared to Reiki and wait-list after 12 weeks (*p* = 0.02; *p* = 0.01) (Beard et al. 2011). As a restriction, it must be mentioned that the Reiki group received an intervention twice a week, whereas the active control group only received an intervention once a week. A sample of breast cancer patients (*n* = 36) showed significantly different developments over the time of four chemotherapy cycles between Reiki, companion and usual care with regard to QoL (*p* < 0.001). The values improved over the study period in both the Reiki arm and the companion arm, and even showed better values for the companion arm over the study period [mean values (standard deviation) at baseline, after chemotherapy 4; Reiki: 103.36 (0.05), 105.53 (0.06); companion: 110.72 (0.06), 114.48 (0.07); usual care: 99.67 (0.06), 98.79 (0.06)] (Orsak et al. 2015). The evaluation of the results in the study is not entirely conclusive; in some cases, fewer or even more participants were evaluated than were actually present in the study group. In cancer patients with polyneuropathy (*n* = 36), no significant effect on QoL in the Reiki, yoga, meditation or education arm was found over 6 weeks (Clark et al. 2012). The study by Catlin and Taylor-Ford (2011) found significant differences in terms of comfort and well-being in favor of the Reiki and placebo arms compared to standard care pre-post chemotherapy (*p* = 0.0197; *p* = 0.002; *p* = 0.0051; *p* = 0.005). However, no significant difference was found between Reiki and placebo arm. All seven studies must be assumed to have an open study design. The second and third study only show a superiority over a resting group. Compared to standard treatments, both Reiki and the sham therapy showed better results (Catlin and Taylor-Ford 2011). No other studies could find any superiority of Reiki; in fact, often the active control treatments (relaxation response therapy, companionship) even delivered better results than Reiki (Beard et al. 2011; Orsak et al. 2015).

Overall, none of the studies provides evidence that Reiki is superior to the comparative active and placebo treatments. Compared to standard treatment (no other support besides normal clinical chemotherapy regime), however, Reiki, active control and placebo treatment showed better short-term results (pre-post chemotherapy) (Catlin and Taylor-Ford 2011). No statement can be made regarding long-term effects, as these were not recorded.

*Neurotoxicity and pain*: the effects of Reiki on neurotoxicity and pain were examined in three RCTs (Clark et al. 2012; Tsang et al. 2007; Olson et al. 2003). Cancer patients with polyneuropathy (*n* = 36) were observed over 6 weeks and no significant differences between the intervention groups (Reiki, yoga, meditation) and passive education group with regard to the symptoms of neurotoxicity were found (Clark et al. 2012). Tsang et al. (2007) observed patients with different types of cancer (*n* = 16) over several weeks and no significant differences between the Reiki group and the rest group regarding pain were found. The endpoint pain was also included in the study by Olson et al. (2003) and an effect of the Reiki interventions was found [significantly better values compared to rest time on day 1 (*p* = 0.035) and on day 4 (*p* = 0.002)](Olson et al. 2003). However, on day 7, no significant difference could be found between the two groups. The study provides little information on blinding and only few statistical parameters. Regarding the effect of Reiki on pain, only one study indicates that Reiki could achieve better pain outcomes compared to a resting group (Olson et al. 2003). In this study, however, positive results were only found on day 1 and day 4; after seven days there were no longer any differences. In none of the other studies could an advantage of Reiki over the comparative treatments be proven (Clark et al. 2012; Tsang et al. 2007). A significant deterioration in neurotoxicity was found in the passive education group, but not in the Reiki group or the active control groups (Clark et al. 2012).

Overall, no study has shown an advantage of Reiki over an active control group regarding neurotoxicity and pain. Differences compared to a passive control group were only short-term effects that were no longer detectable after 7 days.

Side effects: two studies investigated side effects associated with Reiki (Alarcao and Fonseca 2016; Orsak et al. 2015). According to the studies, no side effects were observed. However, the instruments intended for recording side effects are not discussed.

##### Therapeutic Touch

Eight RCTs examined Therapeutic Touch (Aghabati et al. 2010; Frank et al. 2007; Giasson and Bouchard 1998; Matourypour et al. 2015, 2016; Samarel et al. 1998; Tabatabaee et al. 2016; Vanaki et al. 2016). The primary outcomes were anxiety, mood, fatigue, QoL, pain and nausea.

*Anxiety*: the effects of Therapeutic Touch on anxiety were investigated in two RCTs that recorded either anxiety or anxiety-related moods as primary endpoints (Frank et al. 2007; Samarel et al. 1998). In a sample of women with suspected breast cancer (*n* = 82) who received a 10-min intervention (Therapeutic Touch or placebo) during a breast biopsy, no significant difference was found between the arms with regard to anxiety, nervousness, fearfulness, restlessness, tension and fear before and shortly after the intervention (Frank et al. 2007). In addition, no significant differences were found for systolic and diastolic blood pressure and pulse. The number of participants in the study was too small to be able to deliver reliable results and an unblinding cannot be ruled out. The other study examined breast cancer patients (*n* = 35) up to 1 week before an operation and up to one week after discharge from the hospital and recorded state anxiety and trait anxiety of the patients (Samarel et al. 1998). There was a significantly lower preoperative state anxiety in the Therapeutic Touch arm compared to rest with dialogue (*p* = 0.008). Postoperatively, however, there was no significant difference between the arms (*p* = 0.493). Trait anxiety was assessed only once at the beginning of the study or before the first intervention, so no treatment effects were recorded here. The measurement showed no significant difference between the groups (*p* = 0.414). The study did not include a baseline measurement of state anxiety and there was a preselection of the study participants by the surgeons, which may lead to a selection bias. According to the study, some patients were not included because, according to the surgeons, they were too overwhelmed by the diagnosis of breast cancer and too vulnerable to participate in the study. This could have distorted the results, as patients with high anxiety levels at baseline were not included. Furthermore, there may have been a performance bias in the study because Therapeutic Touch was administered together with an interview, so it is not clear whether the effect is due to Therapeutic Touch alone.

Overall, only one out of two studies found a significant improvement in the preoperative state anxiety of patients using Therapeutic Touch in combination with a conversation compared to resting and conversation (Samarel et al. 1998). However, the methodological limitations mentioned above must be taken into account when considering the results.

*Mood*: the effects of Therapeutic Touch on the mood of patients were investigated as the primary endpoint in one study (Samarel et al. 1998). In this study, breast cancer patients (*n* = 35) were examined up to 1 week before surgery and up to 1 week after discharge from hospital. There were no differences between the Therapeutic Touch and rest with dialogue in terms of patient’s mood both preoperatively and postoperatively (*p* = 0.156, *p* = 0.848). Due to the serious methodological limitations of this study, discussed above, the results reported cannot be considered reliable.

Overall, the results do not provide any indication of advantages of Therapeutic Touch over rest with dialogue in terms of patient mood.

*Fatigue*: the endpoint fatigue was considered in the study by Aghabati et al. (2010) over 5 days and a significant reduction was found in female cancer patients (*n* = 90) who were in the Therapeutic Touch group compared to the routine care and the sham group (*p* < 0.001). The blinding is insufficiently described in this study. Therefore, expectancy effects of the study participants cannot be excluded. A missing correction for multiple testing may have led to statistically incorrect significance. Thus, the result is to be classified as not reliable. The baseline comparability is unclear, which greatly limits the results.

Overall, there was a significant improvement of fatigue in the Therapeutic Touch group compared to a sham and routine care group; however, due to the serious methodological limitations, the reported results cannot be considered reliable.

*Quality of life*: Giasson and Bouchard (1998) examined a study group of terminal cancer patients (*n* = 20) regarding QoL and found an effect of Therapeutic Touch (significantly better scores compared to a rest period group on the well-being subscale, *p* = 0.0015). The study showed a low overall study quality. Particularly due to the lack of blinding, the implementation of the intervention by the author of the study and insufficient information on statistical parameters, the result is not reliable.

Therapeutic Touch showed significantly better scores compared to a resting group regarding QoL, but the results are limited by low study quality.

*Pain*: the effects of Therapeutic Touch on pain were examined in four RCTs (Frank et al. 2007; Samarel et al. 1998; Tabatabaee et al. 2016; Aghabati et al. 2010), all of which considered pain as the primary endpoint. Two studies examined a sample of breast cancer patients. The study by Frank et al. (2007) measured the pain before and shortly after a breast biopsy (*n* = 82) and the study by Samarel et al. (1998) measured it 1 week after the patients underwent an operation (*n* = 35). Neither study found a significant advantage for Therapeutic Touch compared to a placebo group or a group that received rest periods combined with conversation (*p* = 0.53, *p* = 0.972). In the first study, the number of participants was too small to be able to deliver reliable results. In the second study, the surgeons pre-selected the study participants, which means that the informative value of the results is not very representative. In another study, the various endpoints impaired by pain (general activity, mood, walking ability, relationship with other people, sleep) of men with different cancer diagnoses (*n* = 90) were investigated over 4 weeks (Tabatabaee et al. 2016). There were significant differences in all endpoints between the Therapeutic Touch group and the placebo group and between the Therapeutic Touch group and the usual care group after 4 weeks (*p* < 0.001). However, there were no significant differences between the placebo group and the usual care group. When looking at the values, it is noticeable that only Therapeutic Touch was able to decrease the pain that affected the ability to walk, whereas the pain in the other two arms worsened. It is not revealed whether the individual study groups were comparable, as no information was given about the cancer diagnoses the patients had or about the treatments they had already undergone. It should be noted that the comparability of the baseline values for pain in the various domains between the groups before the intervention was unclear. Without the comparability given initially, the results of the study cannot be clearly interpreted, as the intervention group could have had better values right from the start. The endpoint pain was considered within the study by Aghabati et al. (2010) over 5 days and significantly lower pain scores were found in female cancer patients (*n* = 90) who were in the Therapeutic Touch group compared to the usual care and the sham group (*p* < 0.001). However, this study shows methodological deficiencies, especially with regard to the comparability of the groups and the trustworthiness of the statistical analyses and their results.

None of the four studies gives any clear indication that there are advantages in reducing pain compared to placebo or comparative treatment. Two studies found no significant differences between the groups (Frank et al. 2007; Samarel et al. 1998). The third study found significant differences with better results in the Therapeutic Touch group regarding various endpoints impaired by pain (general activity, mood, walking ability, relationship with other people, sleep) (Tabatabaee et al. 2016). Also, the study by Aghabati et al. (2010) found an effect of Therapeutic Touch compared to usual care and sham groups. Significant differences were found on each day of a 5-day period. With regard to the effects found, the above-mentioned methodological limitations must be taken into account, so that the reliability is severely limited.

*Nausea*: the effects of Therapeutic Touch on nausea (duration, frequency, intensity and onset) were examined in a RCT, the results of which were partially published in three different publications (Matourypour et al. 2015, 2016; Vanaki et al. 2016). The study included women with breast cancer (*n* = 108) undergoing chemotherapy. A significantly shorter duration of nausea in the Therapeutic Touch arm compared to placebo and standard therapy was found (*p* < 0.001) and no differences were found between placebo and standard therapy (*p* = 0.30) (Matourypour et al. 2015; Vanaki et al. 2016). When looking at the frequency of nausea, the values in the Therapeutic Touch and placebo arm were significantly better than standard therapy (*p* < 0.001). There was also a significant delay in the onset of nausea in the Therapeutic Touch arm compared to the other two arms (*p* < 0.001). Furthermore, the intensity of nausea was measured with a checklist before and after the intervention (Matourypour et al. 2016; Vanaki et al. 2016). After the intervention, significantly better values were again found in the Therapeutic Touch arm and placebo arm compared to standard therapy (*p* < 0.0001). When comparing the Therapeutic Touch arm and the placebo arm, there were no significant differences (*p* = 0.07). Although the three publications have the same sample and examine the same endpoints, the ways in which they are presented differ to the extent that one could assume that they are not the same sample, nor thus the same survey. The initial comparability of the groups is also not shown convincingly in any of the studies. Overall, the credibility and reliability of the results described above are so severely restricted by the serious methodological deficiencies and the pronounced selective reporting that the effects found with regard to bioenergy therapies can be viewed as extremely questionable.

The study suggests advantages of Therapeutic Touch compared to placebo and standard therapy with regard to duration and onset of nausea. However only short-term therapeutic effects (24 h after chemotherapy) were recorded. No statement could be made regarding long-term effects. In terms of frequency and intensity of nausea, significantly better results were found in the Therapeutic Touch group compared to standard therapy, but not compared to placebo. Due to the limitations mentioned above, the study results seem very questionable.

*Side effects*: no study provided information on side effects associated with Therapeutic Touch.

##### Healing Touch

Three RCTs examined Healing Touch (FitzHenry et al. 2014; Lutgendorf et al. 2010; Post-White et al. 2003). The primary outcomes were anxiety, relaxation, depression, fatigue, QoL, pain and nausea.

*Anxiety and relaxation*: the effects of Healing Touch on anxiety and relaxation were examined in one study (Lutgendorf et al. 2010). However, no significant differences were found between Healing Touch, relaxation training and usual care. Furthermore, no group difference was found considering the patient’s blood pressure, which was used as a parameter for relaxation. The small sample size in the study limits this result, otherwise the evidence level of the study was high.

Overall, the results do not provide any indication of advantages of Healing Touch over relaxation training or usual care in terms of anxiety and relaxation.

*Depression*: one study also examined the effects of Healing Touch on depression (Lutgendorf et al. 2010). Patients with cervical cancer (*n* = 60) undergoing radiotherapy were followed for 6 weeks. The first questionnaire (Center for Epidemiological Studies Depression Scale) found no significant improvement over time for Healing Touch compared to relaxation training or usual care. Only for the subscale depressed mood, a significantly larger decrease for Healing Touch was found (Healing Touch vs. relaxation training and usual care *p* = 0.042). There was no significant difference between relaxation training and usual care (*p* = 0.84). Moreover, at the end of the study, no significant difference was found between the arms (*p* = 0.056). For the second questionnaire (Profile of Mood States-Short Form), the authors found a significant difference in the decrease of depression for Healing Touch compared to relaxation training and usual care (*p* = 0.046). Again, there was no significant reduction in the relaxation arm or the usual care arm. As in the first questionnaire, no significant difference was found at the end of the study (*p* = 0.069). While the small sample size in the study limits this result, the methodological quality of the study was high.

Overall, no clear advantage of Healing Touch over relaxation training and standard treatment was shown in terms of improvement of depression.

*Fatigue*: three studies also examined the effects of Healing Touch on fatigue (FitzHenry et al. 2014; Lutgendorf et al. 2010; Post-White et al. 2003). One publication considered the intensity of fatigue and its impact on daily life in breast cancer patients (*n* = 44) after surgery for five to seven weeks (FitzHenry et al. 2014). There were significantly worse values in the Healing Touch compared to the placebo arm over the entire course of the study, due to a non-significant greater improvement in the values in the placebo arm (*p* = 0.024, *p* = 0.010 resp.). No significant differences in intensity of fatigue and impact on daily life were found between the two groups. In another study of patients with cervical cancer (*n* = 60) undergoing radiotherapy, there was no significant difference between Healing Touch, relaxation training and usual care regarding fatigue over 6 weeks (Lutgendorf et al. 2010). The study by Post-White et al. (2003) found an effect of Healing Touch compared to standard care, with fatigue being measured at the beginning of the first and last session of each 4-week crossover period (sessions 1, 4, 5 and 8, *p* = 0.028). However, the results must be considered as being at high risk of bias due to an open study design with possible expectancy effects. Based on the lack of an active comparison group, effects due to increased attention to patients cannot be excluded.

Overall, the studies do not provide clear evidence of a positive effect of Healing Touch on fatigue, especially when compared to a placebo group. Only one study found better results for Healing Touch compared to standard care.

*Quality of life*: the effects of Healing Touch on QoL were investigated in two RCTs (FitzHenry et al. 2014; Lutgendorf et al. 2010). One study examined breast cancer patients (*n* = 44) after surgery for five to seven weeks (FitzHenry et al. 2014). There were no significant differences in QoL between the Healing Touch and placebo arm. In the other study, patients with cervical cancer (*n* = 60) undergoing radiotherapy were examined for 6 weeks (Lutgendorf et al. 2010). There was no significant difference in QoL between Healing Touch, relaxation training and usual care. Only the small sample size in both studies limits this result, otherwise the evidence level in the studies was high.

Concerning Healing Touch, the two studies found no benefits in terms of QoL compared to placebo and relaxation therapy and compared to usual care.

*Pain*: The endpoint pain was considered in the study by Post-White et al. (2003) and no advantage for Healing Touch regarding pain was found compared to the caring presence group and compared to massage therapy over 4 weeks. Overall, the publication was a high-quality, large-scale study.

Overall, the results do not provide any indication of advantages of Healing Touch over caring presence group or massage therapy in terms of pain.

*Nausea*: the study by Post-White et al. (2003) also examined the endpoint nausea and found no significant differences between Healing Touch, massage and caring presence. Nausea data were measured just prior to and just after each weekly session over 4 weeks. The quality of the study was high, and it was a large-scale study.

Overall, the results show no advantage of Healing Touch compared to massage and caring presence in terms of nausea.

*Side effects*: no study provided information on side effects associated with Healing Touch.

##### Polarity Therapy

Two RCTs examined Polarity Therapy (Mustian et al. 2011; Roscoe et al. 2005). The primary outcome was fatigue and QoL.

*Fatigue*: fatigue in breast cancer patients (*n* = 45), most of whom had already undergone surgery and chemotherapy and were currently undergoing radiotherapy, was assessed over 3 weeks using a questionnaire (BFI) and patient diaries (Mustian et al. 2011). No significant differences were found between the three arms (Polarity Therapy, massage, standard care) by the questionnaire (*p* = 0.72). Analysis of the diaries showed no significant differences in fatigue scores between Polarity Therapy and massage when baseline values were considered. The other study also considered breast cancer patients (*n* = 16) undergoing radiotherapy (Roscoe et al. 2005). The observation period was 2 weeks and one Polarity Therapy group received one treatment season and the other Polarity Therapy group received two treatment seasons. After 1 week, the Polarity Therapy groups showed significantly better values for fatigue compared to standard care (*p* = 0.04). After the second week the authors described significantly better values in the Polarity Therapy groups compared to standard care. Yet, the *p*-value is 0.05. There were no significant differences between the two polarity groups. Both studies have a low number of participants.

For Polarity Therapy, no advantage in terms of fatigue compared to massage and standard treatment was found (Mustian et al. 2011). The study by Roscoe et al. (2005) found significantly better values for fatigue in the Polarity Therapy groups compared to standard care. Regarding the study quality, it should be said that this was a very small study population (five participants evaluated in each arm). Furthermore, an active comparison group is missing, so that expectation and attention effects cannot be excluded. Moreover, the data collection took place at different timepoints (depending on the patient, from Tuesday to Friday evening after the intervention), which further limits the comparability of the results.

*Quality of life*: the effects of Polarity Therapy on QoL were investigated in two RCTs (Mustian et al. 2011; Roscoe et al. 2005). Mustian et al. (2011) examined breast cancer patients (*n* = 45), most of whom had already undergone surgery and chemotherapy and were currently undergoing radiotherapy for 3 weeks. There were no significant differences in QoL between Polarity Therapy, massage and standard care (*p* = 0.21). The study also collected patient feedback on the Polarity Therapy and massage therapy after 3 weeks. Both treatments were considered recommendable by the patients with a slightly higher recommendation rate in the massage group (no information on significance). Another study also considered breast cancer patients (*n* = 16) undergoing radiotherapy (Roscoe et al. 2005). The observation period was 2 weeks, and the patients received a Polarity Therapy application once a week, in one group for one week and in another group for 2 weeks. After 1 week, the Polarity Therapy groups showed significantly better QoL values compared to standard care (*p* = 0.02). After the second week, however, no significant differences between the groups could be detected (*p* = 0.68). Due to the open study design and the small number of participants, the results presented are only conditionally reliable.

Overall, only one study found a short-term (three days after first intervention) significant positive effect of Polarity Therapy compared to standard care in terms of QoL (Roscoe et al. 2005). In the long term (2 weeks after the start of the study), however, no differences between the groups were observed.

*Side* *effects*: one study investigated side effects associated with Polarity Therapy (Roscoe et al. 2005). According to the study, no side effects were observed. However, the instruments intended for recording side effects were not discussed.

## Discussion

In our systematic review, we were able to include 21 RCTs with 1375 patients. Yet, despite this rather large number of studies, there is no evidence of a positive effect of bioenergy therapies in cancer care. This is mainly due to the low quality of all RCTs. The selection of the comparator in the control group is a decisive criterion for the validity of the result. Most studies only have a passive control group. Accordingly, in contrast to the active bioenergy therapies groups, attention effects may strongly affect the results. In the comparisons with an active control group, for example a sham group, no effects were detectable, which also supports the hypothesis of attention effects.

Only three of the studies examined are of high quality (FitzHenry et al. 2014; Lutgendorf et al. 2010; Post-White et al. 2003). However, two of these studies (FitzHenry et al. 2014; Lutgendorf et al. 2010) only have a small number of study participants (*n* = 41, *n* = 51), so that the representativeness and informative value is rather low.

In all other studies, the most important drawback is that the studies have an open design without adequate blinding or without information on blinding. Furthermore, some studies lack data on the initial comparability of the study groups and their baseline values. Another (possible) limitation of the included studies is the different scale systems for grading the treatment toxicities. After considering the methodological quality of the studies, Reiki, Therapeutic Touch, Healing Touch and Polarity Therapy were not effective for improving anxiety, depression, mood disturbances, fatigue, QoL, neurotoxicity, pain and nausea in patients with cancer.

Considering anxiety, three studies assessed Reiki (Beard et al. 2011; Potter 2007; Tsang et al. 2007), two Therapeutic Touch (Frank et al. 2007; Samarel et al. 1998) and one Healing Touch (Lutgendorf et al. 2010). The validity of the results is severely limited by the open study design and the lack of representativeness of the study population. Only the study assessing Healing Touch had a high quality (Lutgendorf et al. 2010). Accordingly, none of the studies provides evidence that Reiki is superior to the other active and passive treatments. When looking at patients classified as suffering from anxiety at the start of the study, a significant improvement was only found in a RRT group, but not in the Reiki group. Only one out of two studies found a significant improvement in the preoperative state anxiety of patients using Therapeutic Touch in combination with a conversation compared to resting and conversation (Samarel et al. 1998). However, the methodological limitations mentioned above must be taken into account when considering the results. No significant differences were found between Healing Touch, relaxation training and usual care.

Considering depression, two studies assessed Reiki (Beard et al. 2011; Potter 2007) and one Healing Touch (Lutgendorf et al. 2010). All studies had a small sample size, which limits the results. Only the study regarding Healing Touch had a high quality (Lutgendorf et al. 2010). When looking at the effects of Reiki on depression, only one study found a significant improvement in patients who were classified as depressed at the start of the study (Beard et al. 2011). However, the same significant results were detectable in the RRT and wait-list group. Overall, no clear advantage of Healing Touch over relaxation training and standard treatment was shown in terms of improvement of depression.

Considering mood disturbances, two studies assessed Reiki (Clark et al. 2012; Orsak et al. 2015) and one Therapeutic Touch (Samarel et al. 1998). Lack of blinding, selection bias, and performance bias limit the validity of the results. One of the two examined studies showed evidence that Reiki was superior to usual care, but not in comparison to other therapies such as yoga, relaxation, or companionship, which means that the results could at least largely be due to attention effects. Overall, neither study provides evidence for the benefits of Reiki beyond placebo in terms of improving mood disturbances and the results do not provide any indication of advantages of Therapeutic Touch over rest with dialogue in terms of patient mood.

Considering fatigue, one study assessed Reiki (Tsang et al. 2007), one study Therapeutic Touch (Aghabati et al. 2010), three studies Healing Touch (FitzHenry et al. 2014; Lutgendorf et al. 2010; Post-White et al. 2003) and two studies Polarity Therapy (Mustian et al. 2011; Roscoe et al. 2005). The results must be considered as impaired because of an open study design with possible expectancy and attention effects. Accordingly, the available evidence gives no indication of a positive effect of Reiki on fatigue. Regarding the significant improvement of fatigue in the Therapeutic Touch group compared to a sham and routine care group, expectancy effects of the study participants cannot be excluded due to an inadequately described blinding. Only one of the three studies found better results for Healing Touch compared to standard care regarding fatigue. The results must be considered as impaired because of an open study design with possible expectancy effects. The study situation gives no indication of a positive effect of Polarity Therapy compared with an active control group regarding fatigue, so the effect compared with standard care may be due, at least in large part, to attentional or expectancy effects.

Considering QoL seven studies assessed Reiki (Alarcao and Fonseca 2016; Beard et al. 2011; Catlin and Taylor-Ford 2011; Clark et al. 2012; Orsak et al. 2015; Tsang et al. 2007; Olson et al. 2003), one Therapeutic Touch (Giasson and Bouchard 1998), two Healing Touch (FitzHenry et al. 2014; Lutgendorf et al. 2010) and two Polarity Therapy (Mustian et al. 2011; Roscoe et al. 2005). Most of the studies had low quality due to open design, lack of placebo group, and small number of participants. Therefore, neither attention effects nor expectancy effects can be excluded. Only the two studies assessing Healing Touch had high quality (FitzHenry et al. 2014; Lutgendorf et al. 2010). None of the studies provides evidence that Reiki is superior to the comparative active and placebo treatments. Compared to standard treatment (no other support besides normal clinical chemotherapy regime), however, Reiki, active control and placebo treatment showed better short-term results (pre-post chemotherapy) (Catlin and Taylor-Ford 2011). No statement can be made regarding long-term effects, as these were not recorded. No other studies could find any superiority of Reiki, and often even the active control treatments (relaxation response therapy, companionship) delivered better results than Reiki (Beard et al. 2011; Orsak et al. 2015). Regarding the effect of Therapeutic Touch, significantly better scores compared to a resting group were found (Giasson and Bouchard 1998), but the study showed a low overall study quality. Concerning Healing Touch, the two studies found no benefits in terms of QoL compared to placebo and relaxation therapy and compared to usual care. When looking at Polarity Therapy, only one study found a short-term (three days after first intervention) significant positive effect of Polarity Therapy compared to standard care in terms of QoL (Roscoe et al. 2005). The study quality must be classified as low, since neither attention effects nor expectancy effects can be excluded.

Considering neurotoxicity and pain, three studies assessed Reiki (Clark et al. 2012; Tsang et al. 2007; Olson et al. 2003), four Therapeutic Touch (Frank et al. 2007; Samarel et al. 1998; Tabatabaee et al. 2016; Aghabati et al. 2010) and one Healing Touch (Post-White et al. 2003). The results must be critically questioned because most of the studies were of low quality. It is not clear whether the individual groups were comparable at all and whether the bioenergy groups already had better values than the other groups at the beginning. Only the study assessing Healing Touch had high quality (Post-White et al. 2003). No study has shown an advantage of Reiki over an active control group regarding neurotoxicity and pain. Differences compared to a passive control group were only short-term effects that were no longer detectable after seven days. Considering the effects of Therapeutic Touch regarding pain, although two of the studies provide evidence of an advantage of Therapeutic Touch over both placebo and passive control, the study quality was low. When looking at Healing Touch and pain, no significant differences between Healing Touch, massage and caring presence were found in one high-quality study.

Considering nausea, one study assessed Therapeutic Touch (Matourypour et al. 2015, 2016; Vanaki et al. 2016) and one Healing Touch (Post-White et al. 2003). The credibility and reliability of the study investigating Therapeutic Touch are so severely limited by serious methodological deficiencies and pronounced selective reporting that the results must be regarded as extremely questionable (Matourypour et al. 2015, 2016; Vanaki et al. 2016). The study regarding Healing Touch was a high quality, large-scale study (Post-White et al. 2003). The study with low quality suggests benefits of Therapeutic Touch compared to placebo and passive control treatment. The result of the high-quality study shows no advantage of Healing Touch compared to massage and caring presence in terms of nausea.

To summarize, studies with high study quality could not find any difference between bioenergy therapies and active (placebo, massage, RRT, yoga, meditation, relaxation training, companionship, friendly visit) and passive control groups (usual care, resting, education). Only studies with a low study quality were able to show significant effects. Overall, the reliability of the results is severely limited by open study design. In particular, in some studies attention and expectation effects, due to the lack of a placebo group and the participation of test persons who showed a special motivation, cannot be ruled out or may be regarded as likely. Therefore, the result could be considered as distorted.

### Limitations of this work

There are some limitations to this systematic review. First, we excluded studies concerning children or teenagers. Furthermore, only studies in English or German language were included. Since only RCTs were included in the review, the total number of studies is limited. However, including other study types would not have added high quality evidence. Another (possible) limitation of this study is the different scale systems for grading the treatment toxicities.

## Conclusions

In the overall evaluation of bioenergy therapies, no advantage over placebo, massage, relaxation response therapy, yoga, meditation, relaxation training, companionship, friendly visit, resting, education and usual care was found. All in all, it can be assumed that the positive effects were only due to attention and expectation effects. The review also found no evidence that trained Reiki therapists have an advantage over sham therapy. Data on side effects are only mentioned in three studies (Alarcao and Fonseca 2016; Orsak et al. 2015; Roscoe et al. 2005). However, the instruments intended for recording side effects are not discussed. None of the studies could find relevant side effects.

## Supplementary Information

Below is the link to the electronic supplementary material.

Supplementary file1 (DOCX 24 KB)

Supplementary file2 (DOCX 34 KB)

Supplementary file3 (DOCX 23 KB)

Supplementary file4 (DOCX 181 KB)

Supplementary file5 (DOCX 31 KB)
