# Supplementary material for: Bioenergy therapies as a complementary treatment: a systematic review to evaluate the efficacy of bioenergy therapies in relieving treatment toxicities in patients with cancer
Source: J Cancer Res Clin Oncol. 2022 Sep 27;149(6):2607–19. doi: 10.1007/s00432-022-04362-x (PMC10129966; doi:10.1007/s00432-022-04362-x)
Supplement: Supplementary file 1 — Supplementary file1 (DOCX 24 KB) [file 432_2022_4362_MOESM1_ESM.docx]

Search string

| Database | Search strategy |
| --- | --- |
| OVID Medline | 1 Therapeutic Touch/ or reiki.mp. or rei-ki.mp. or therapeutic touch.mp. or polarity therap$.mp. or healing touch.mp.  2 exp neoplasms/ or neoplasm$.mp or cancer$.mp. or tumo?r$.mp. or malignan$.mp. or oncolog$.mp. or carcinom$.mp. or leuk?emia.mp. or lymphom$.mp. or sarcom$.mp. or preneoplas$.mp. or exp Precancerous Conditions/ or precancer$.mp.  3 1 AND 2  4 limit 3 to english or limit 3 to german  5 limit 4 to yr="1995 -Current"  6 (5 and humans/) or (5 not animals/)  7 (((comprehensive* or integrative or systematic*) adj3 (bibliographic* or review* or literature)) or (meta-analy* or metaanaly* or "research synthesis" or ((information or data) adj3 synthesis) or (data adj2 extract*))).ti,ab. or (cinahl or (cochrane adj3 trial*) or embase or medline or psyclit or (psycinfo not "psycinfo database") or pubmed or scopus or "sociological abstracts" or "web of science" or central).ab. or ("cochrane database of systematic reviews" or evidence report technology assessment or evidence report technology assessment summary).jn. or Evidence Report: Technology Assessment*.jn. or network adj1 analy*.ti,ab.) or (((review adj5 (rationale or evidence)).ti,ab. and review.pt.) or meta-analysis as topic/ or Meta-Analysis.pt.)  8 Randomized controlled trial.pt. or controlled clinical trial.pt. or randomi?ed.ti,ab.or placebo.ti,ab. or drug therapy.sh. or randomly.ti,ab. or trial?.ti,ab. or group?.ti,ab.  9 6 AND (7 OR 8) |
| OVID Embase | 1 reiki/ or reiki.mp. or rei-ki.mp. or therapeutic touch.mp. or polarity therap$.mp. or heal$ touch.mp  2 exp neoplasms/ or neoplasm$.mp or cancer$.mp. or tumo?r$.mp. or malignan$.mp. or oncolog$.mp. or carcinom$.mp. or leuk?emia.mp. or lymphom$.mp. or sarcom$.mp. or preneoplas$.mp. or exp Precancerous Conditions/ or precancer$.mp.  3 1 AND 2  4 limit 3 to english or limit 3 to german  5 limit 4 to yr="1995 -Current"  6 (5 and humans/) or (5 not animals/)  7 ((((comprehensive* or integrative or systematic*) adj3 (bibliographic* or review* or literature)) or (meta-analy* or metaanaly* or "research synthesis" or ((information or data) adj3 synthesis) or (data adj2 extract*))).ti,ab. or (cinahl or (cochrane adj3 trial*) or embase or medline or psyclit or (psycinfo not "psycinfo database") or pubmed or scopus or "sociological abstracts" or "web of science" or central).ab. or ("cochrane database of systematic reviews" or evidence report technology assessment or evidence report technology assessment summary).jn. or Evidence Report: Technology Assessment*.jn. or (network adj1 analy*).ti,ab.) or (exp Meta Analysis/ or ((data extraction.ab. or selection criteria.ab.) and review.pt.))  8 crossover procedure/ or double blind procedure/ or randomized controlled trial/ or single blind procedure/ or (random$ or factorial$ or crossover$ or (cross adj1 over$) or placebo$ or (doubl$ adj1 blind$) or (singl$ adj1 blind$) or assign$ or allocat$ or volunteer$).ti,ab,de.  9 5 AND (6 OR 7) |
| Cochrane | #1 [mh “Therapeutic Touch”] or reiki or rei-ki or “therapeutic touch” or (polarity NEXT therap*) or (heal* NEXT touch)  #2 [mh neoplasms] or neoplasm* or cancer? or tum*r? or malignan* or oncolog* or carcinom* or leuk*mia or lymphoma? or sarcoma? or precancer* or preneoplas*  #3 #1 AND #2 |
| EBSCO PsychINFO | S1 reiki or rei-ki or “therapeutic touch” or “polarity therap*” or “heal* touch”  S2 ((DE "Neoplasms" OR DE "Benign Neoplasms" OR DE "Breast Neoplasms" OR DE "Endocrine Neoplasms" OR DE "Leukemias" OR DE "Melanoma" OR DE "Metastasis" OR DE "Nervous System Neoplasms" OR DE "Terminal Cancer") OR  (TX neoplasm* OR TX cancer OR TX tumo#r OR TX malignan* OR DE „oncology“ OR TX oncolog* OR TX carcinom* OR TX leuk#emia OR TX lymphoma OR TX sarcoma OR TX precancer* OR TX preneoplas*))  S3 LA (English OR German)  S4 S1 AND S2 AND S3  S5 ((comprehensive* OR integrative OR systematic*) N3 (bibliographic* OR review* OR literature)) OR (meta-analy* or metaanaly* or "research synthesis" OR ((information OR data) N3 synthesis) OR (data N2 extract*)) OR ((review N5 (rationale OR evidence)) AND DE "Literature Review") OR (AB(cinahl OR (cochrane N3 trial*) OR embase OR medline OR psyclit OR pubmed OR scopus OR "sociological abstracts" OR "web of science" OR central)) OR DE "Meta Analysis" OR (network N1 analy*)  S6 DE "Treatment Effectiveness Evaluation" OR DE "Treatment Outcomes" OR DE "Psychotherapeutic Outcomes" OR DE "Placebo" or DE "Followup Studies" OR placebo* OR random* OR "comparative stud*" OR (clinical N3 trial*) OR (research N3 design) OR (evaluat* N3 stud*) OR (prospectiv* N3 stud*) OR ((singl* OR doubl* OR trebl* OR tripl*) N3 (blind* OR mask*)  S7 S4 AND (S5 OR S6) |
| EBSCO Cinahl | S1 MH “healing touch” or MH Reiki or MH “Therapeutic Touch” or MH “Polarity Therapy” or reiki or rei-ki or “therapeutic touch” or “heal* touch” or “polarity therap*”  S2 (MH "Neoplasms+" OR TX neoplasm* OR TX cancer OR TX tumo#r OR TX malignan* OR TX oncolog* OR TX carcinom* OR TX leuk#emia OR TX lymphoma OR TX sarcoma OR MH "Precancerous Conditions+" OR TX precancer* OR TX preneoplas*)  S3 (LA German OR LA English)  S4 S1 AND S2 AND S3  S5 (TI (systematic* n3 review*)) or (AB (systematic* n3 review*)) or (TI (systematic* n3 bibliographic*)) or (AB (systematic* n3 bibliographic*)) or (TI (systematic* n3 literature)) or (AB (systematic* n3 literature)) or (TI (comprehensive* n3 literature)) or (AB (comprehensive* n3 literature)) or (TI (comprehensive* n3 bibliographic*)) or (AB (comprehensive* n3 bibliographic*)) or (TI (integrative n3 review)) or (AB (integrative n3 review)) or (JN “Cochrane Database of Systematic Reviews”) or (TI (information n2 synthesis)) or (TI (data n2 synthesis)) or (AB (information n2 synthesis)) or (AB (data n2 synthesis)) or (TI (data n2 extract*)) or (AB (data n2 extract*)) or (TI (medline or pubmed or psyclit or cinahl or (psycinfo not “psycinfo database”) or “web of science” or scopus or embase)) or (AB (medline or pubmed or psyclit or cinahl or (psycinfo not “psycinfo database”) or “web of science” or scopus or embase or central)) or (MH “Systematic Review”) or (MH “Meta Analysis”) or (TI (meta-analy* or metaanaly*)) or (AB (meta-analy* or metaanaly*)) or TI (network analy*) or AB (network analy*)  S6 (MH "Clinical Trials+") or PT Clinical trial or TX clinic* n1 trial* or TX ( (singl* n1 blind*) or (singl* n1 mask*)) or TX ((doubl* n1 blind*) or (doubl* n1 mask*)) or TX ( (tripl* n1 blind*) or (tripl* n1 mask*)) or TX ((trebl* n1 blind*) or (trebl* n1 mask*)) or TX randomi* control* trial* or (MH "Random Assignment") or TX random* allocat* or TX placebo* or MH "Placebos") or MH "Quantitative Studies") or TX allocat* random*  S7 S4 AND (S5 OR S6) |
