# Supplementary material for: Bioenergy therapies as a complementary treatment: a systematic review to evaluate the efficacy of bioenergy therapies in relieving treatment toxicities in patients with cancer
Source: J Cancer Res Clin Oncol. 2022 Sep 27;149(6):2607–19. doi: 10.1007/s00432-022-04362-x (PMC10129966; doi:10.1007/s00432-022-04362-x)
Supplement: Supplementary file 2 — Supplementary file2 (DOCX 34 KB) [file 432_2022_4362_MOESM2_ESM.docx]

Table 1. Characterization of the included studies

| Reference | Study  type | n/  cancer  type/  Drop  Out | Intervention/  Duration | Endpoints | Outcomes |
| --- | --- | --- | --- | --- | --- |
| Aghabati (2010) | Randomized controlled study | n=90  No specific indication of tumor type  No information about Attrition and Drop-Out  Evaluated: N/A | Arm A:  N=30  Therapeutic Touch  30 min per day over 5 days  Arm B: N=30  Sham Therapeutic Touch  30 min per day over 5 days  Arm C:  N=30  Routine care | 1. Pain with VAS | 1. Comparison of arms: on 5 of 5 days significant reduction in TT compared to sham and control arm, p< 0.001 on first day of treatment |
| Alarcão (2016) | Randomized controlled study | n=116  Hematological cancer  Attrition=16 (deceased)  Drop-Out: N/A  Evaluated: n=100 | Arm A:  N=58  Reiki therapy 2x 60 min per week for 4 weeks  Arm B:  N=58  Placebo (sham) Reiki therapy  2x 60 min per week for 4 weeks | 1. Physical domain of WHOQoL-100/BREF  2. Psychological domain of WHOQoL-100/BREF  3. Social relationships domain of WHOQoL-100/BREF  4. Environment domain of WHOQoL-100/BREF  5. Score for each domain of WHOQoL-100/BREF | 1. Comparison of both arms: significantly higher values in Reiki compared to sham arm, p= 0.015  2. Comparison of both arms: no significant differences between Reiki and sham arm, p= 0.186  3. Comparison of both arms: significantly higher values in Reiki compared to sham arm, p= 0.0005  4. Comparison of both arms: significantly higher values in Reiki compared to sham arm, p= 0.0075  5. Comparison of both arms: significantly higher values in Reiki compared to sham arm, p= 0.035 |
| Beard (2011) | Randomized controlled study | n=54  Prostate cancer  Attrition=0  Drop-Out=5  Evaluated: n=54 (Intention-to-treat) | Arm A:  N=18  Reiki therapy 2x 50 min per week for 8 weeks  Arm B:  N=18  RRT/Cognitive Restructuring (CR) 1x 60 min per week for 8 weeks + recommendation for daily exercise + documentation in a diary at home  Arm C:  N=18  Control arm, waiting list (with the option to receive either Reiki or RRT / CR at the end of the study) | 1. Quality of life with FACT-G  2. Emotional well-being subscale of FACT-G | 1. Comparison of arms: no significant differences between Reiki, RRT/CR and control arm, p= N/A  2. Comparison of arms: significantly higher levels of emotional well-being in RRT/CR compared to Reiki (p= 0.02) and control arm (p= 0.01) |
| Catlin (2011) | Randomized controlled study | n=189  No specific indication of tumor type  No information about Attrition and Drop-Out  Evaluated: N/A | Arm A:  N=63  Reiki therapy  1x 20 minutes  Arm B:  N=63  Placebo (sham) Reiki therapy  1x 20 minutes  Arm C:  N=63  Control arm, standard care | 1. Comfort with HTCQ  2. Well-Being with Well-Being Analog Scale  3. Physical well-being (calculated from Well-Being Analog Scale)  4. Mental well-being (calculated from Well-Being Analog Scale)  5. Physical comfort (calculated from HTCQ)  6. Mental comfort (calculated from HTCQ) | 1. Comparison of arms: significant improvement in Reiki (p= 0.0197) and sham (p= 0.0027) compared to control arm; no significant difference between Reiki and sham arm, p= 0.8435  2. Comparison of arms: significant improvement in Reiki (p= 0.0051) and sham (p= 0.005) compared to control arm; no significant difference between Reiki and sham arm, p= 0.7453  3. Comparison of arms: no significant differences between Reiki, sham and control arm, p= N/A  4. Comparison of arms: significant improvement in Reiki (MD= 6.88) and placebo (MD= 6.34) compared to control arm, p< 0.05  5. Comparison of arms: significant improvement in sham (MD= 3.31) compared to control arm, p< 0.05  6. Comparison of arms: significant improvement in Reiki (MD= 3.96) and sham (MD= 3.95) compared to control arm, p< 0.05 |
| Clark (2012) | Randomized controlled study | n=36  Various cancer diagnoses  (65.4% breast carcinoma), stage I-IV  Attrition=10 (Arm A: 2x, Arm B: 2x, Arm C: 4x, Arm D: 2x)  Drop-Out=14  Evaluated: n=26 | Arm A:  N=9  Reiki  1x 60 min per week for 6 weeks + recording of symptoms in a diary  Arm B:  N=9  Yoga  1x 60 min per week for 6 weeks + Daily yoga exercises + Diary of exercises  Arm C:  N=9  Meditation  1x 60 min per week for 6 weeks + Daily meditation exercises + Diary about experiences with exercises  Arm D:  N=9  Control arm, holistic education of chemotherapy induced peripheral neuropathy  1x 60 min per week for 6 weeks + Documentation of follow-up of teaching materials | 1. Quality of life with FACT & GOG-Ntx  2. Neurotoxicity with FACT & GOG-Ntx | 1. ANOVA: No effects of Reiki, Yoga and Meditation, p= 0.693  2. ANOVA: No effects of Reiki, Yoga and Meditation, p= 0.529; significantly worse results in control arm after 6 weeks, p= 0.034 |
| FitzHenry (2014) | Randomized controlled study | n=44  Breast carcinoma (I,II)  Attrition=3  Drop-Out: N/A Evaluated: n=41 | Arm A:  N=21  Healing Touch 1x 45 min per week for 5 to 7 weeks  Arm B:  N=20  Sham Therapy  1x 45 min per week for 5 to 7 weeks | 1. Quality of life with FACT-B including FACT-G and breast-specific subscale | 1. Comparison of both arms: no significant differences between HT and sham arm, p= N/A |
| Frank (2007) | Randomized controlled study | n=82  Suspicious breast lesions, confronted with stereotactic core biopsy  Attrition=0  Drop-Out=0  Evaluated: n=82 | Arm A:  N=42  Krieger-Kunz Therapeutic Touch 1x 10 min  Arm B:  N=40  Placebo (sham) Therapeutic Touch 1x 10 min | 1. Pain with VAS | 1. Comparison of both arms: no significant differences between TT and sham arm, p= 0.53  Subgroup analysis: patients with pain at baseline had lower pain after intervention while patients without pain at baseline had increased pain after intervention, SD= -11.2 ± 38.9 mm vs. 17.2 ± 27.3 mm, p= 0.0007 |
| Giasson and Bouchard (1998) | Randomized controlled study | n=20  Terminal cancer, main primary cancer site: lung  Attrition=0  Drop-Out=0  Evaluated: n=20 | Arm A:  N=10  Therapeutic Touch  3 x 15–20 min for  4 days  Arm B:  N=10  Rest period  3 x 15–20 min for  4 days | 1. Well-Being and Quality of life with POMS, VAS and SF-36 | 1. Significant improvement in well-being of the TT arm after 3 treatments, Mean increase= 1.7, SD= 1.28; p= 0.0015 |
| Lutgendorf (2010) | Randomized controlled study | n=60  Cervical squamous cell carcinoma or adenocarcinoma (IB1-IVA)  Attrition=9 (Arm A: 4x, Arm B: 3x, Arm C: 2x)  Drop-Out: N/A  Evaluated: n=51 | Arm A:  N=21  Healing Touch 4x 20-30 min per week  Arm B:  N=20  Relaxation Training  4x 20-25 min per week  Arm C:  N=19  Control arm, usual care, neutral video during intervention period  Duration: 6 weeks | 1. Quality of life with FACT | 1. Comparison of arms: no significant differences between arms, p= N/A |
| Matourypour (2015)  Matourypour (2016)  Vanaki (2016) | Randomized controlled study | n=108  Breast carcinoma (No metastases to the central nervous system and digestive system)  Attrition=0  Drop-Out=0  Evaluated: n=108 | Arm A:  N=36  Therapeutic Touch  1x 15-20 min shortly before chemotherapy  Arm B:  N=36  Placebo therapy  1x 15-20 min shortly before chemotherapy  Arm C:  N=17  Control arm | 1. Duration of nausea to T1 with acute phase checklist  2. Frequency of nausea to T1 with acute phase checklist  3. Onset of nausea at T1 with acute phase checklist  1. Intensity of vomiting with vomiting intensity scale  1. Duration of nausea with nausea chart of acute phase  2. Frequency of nausea with nausea chart of acute phase  3. Intensity of vomiting (unclear survey) | 1. Comparison of arms: significant lower duration in TT compared to placebo and control arm, p< 0.001; no significant differences between placebo and control arm, p= 0.3  2. Comparison of arms: significant decrease in TT and placebo compared to control arm, p< 0.001  3. Comparison of arms: significant delay in TT compared to placebo and control arm, p< 0.001  1. Comparison of arms: significant reduction in TT and placebo compared to control arm, p< 0.0001; no significant differences between TT and placebo arm, p= 0.07  1. Comparison of arms: significant lower duration in TT compared to placebo and control arm, p< 0.001; no significant differences between placebo and control arm, p= 0.3  2. Comparison of arms: significant decrease in TT and placebo compared to control arm, p< 0.001  3. Kruskal-Wallis-Test: significant group difference, p< 0.001; significant lower intensity in TT arm (not clear if compared to placebo or control arm, p= N/A) |
| Mustian (2011) | Randomized controlled study | n=45  Breast carcinoma (stage 0-III)  Attrition =2 (voluntarily recalled because not enough time)  Drop-Out =3 (1x no data for T2 and T3, 1x no fatigue diary, 1x partial diary data)  Evaluated: n=43 | Arm A:  N=13  Standard clinical care + Polarity Therapy  1x 75 min per week (Monday or Tuesday) for 3 weeks.  Arm B:  N=15  Standard clinical care + modified (swedish) massage  1x 75 min per week (Monday or Tuesday) for 3 weeks  Arm C:  N=15  Control arm with offer to receive 2 massages after the study, standard care | 1. HRQL with FACIT-F  2. Feedback with one question | 1. Comparison of arms: no significant differences between arms, p= 0.21  2. Comparison of arms: Increase in PT and massage arm, p= N/A |
| Olson (2003) | Randomized controlled study | n=73  No specific indication of tumor type  Attrition=20 (only interested in participating Reiki treatments  Drop-Out=29 (5 deaths, 14 withdrawals by research nurse due  to drop in mini-mental status, 3 withdrawals by  patient due to deterioration in health status,  no reason stated for 7 remaining withdrawals)  Evaluated: n=24 | Arm A:  N=13  Opioid plus rest time 1.5 h x 2 sessions  Arm B:  N=11  Opioid plus Reiki therapy  1.5 h x 2 sessions | 1. Pain with VAS  2. Well-Being and Quality of life with POMS, VAS and SF-36  3. Physical indicators | 1. Comparison of both arms: significant reduction for two out of five days in Reiki compared to control arm, p= 0.002 on day 4; no significant differences between Reiki and control arm 3 days later, p= N/A  2. Comparison of both arms: significant improvement in psychological component of quality of life in Reiki (pretest score: 5.4, posttest score: 6.2) compared to control arm, p= 0.002  3. Comparison of both arms: no significant differences between Reiki and control arm, p= N/A |
| Orsak (2015) | Randomized controlled study | n=36  Breast carcinoma (I-III)  Attrition=1 (Arm B)  Drop-Out=2 (Arm A)  Evaluated: N/A | Arm A:  N=15  Reiki therapy 4x 30 min  Arm B:  N=11  Companion  4x 30 min  Arm C:  N=10  Control arm, usual care  Duration: 4 CTX cycles | 1. Quality of life with FACT: Breast Cancer Version 4 | 1. Significant difference between the groups over time, p< 0.001; improvement in Reiki and companion compared to control arm over the study period; better values in companion compared to Reiki arm during the study period |
| Post-White (2003) | Randomized controlled study | n=230  No specific indication of tumor type  Attrition=66 (Arm A: 15x, Arm B: 21x, Arm C: 30x)  Drop-out: N/A  Evaluated: n=164 | Arm A:  N=63  Therapeutic massage  4 x 45 min weekly + 4x control (standard care)  Arm B:  N=56  Healing Touch  4 x 45 min weekly + 4x control (standard care)  Arm C:  N=45  Caring presence 4 x 45 min weekly + 4x control (standard care) | 1. Pain with VAS  2. Nausea with POMS  3. Well-Being and Quality of life with POMS, VAS and SF-36  4. Physical indicators | 1. Comparison of arms: no significant differences between HT, TM and control arm, p= N/A  2. No effects of HT, TM and presence, p= N/A  3. No significant improvement in mood after 4 sessions HT, p= N/A  4. Comparison of arms: no significant differences between HT, TM and control arm, p= N/A |
| Potter (2007) | Randomized controlled study | n=35  Registered for  outpatient breast biopsy  Attrition=3 (No biopsy: Arm A: 1x, Arm B: 2x)  Drop-Out=1 (Arm A: first intervention only because she felt uncomfortable)  Evaluated: n=32 | Arm A:  N=18  Reiki therapy  2x 45-50 min  One within 7 days before and one within 7 days after biopsy  Arm B:  N=17  Control arm, conventional care | 1. Anxiety with STAI  2. Depression with CES-D  3. Anxiety and Depression with HADS  4. Anxiety with HAS subscale  5. Depression with HAS subscale | 1. Comparison of both arms: no significant differences between Reiki and control arm, p= N/A; significant decrease over time in Reiki and control arm, p= 0.0119  2. Comparison of both arms: no significant differences between Reiki and control arm, p= N/A; no significant change over time in Reiki and control arm, p= 0.6200  3. Comparison of both arms: no significant differences between Reiki and control arm, p= N/A; significant decrease over time in Reiki and control arm, p= 0.0187  4. Comparison of both arms: significant decrease over time in Reiki and control arm, p= 0.0011  5. Comparison of both arms: no significant change over time in Reiki and control arm, p= 0.7023 |
| Roscoe (2005) | Randomized controlled study | n=16  Breast Cancer  Attrition=1 (Data not to be used)  Drop-Out: N/A  Evaluated: n=15 | Arm A:  N=5  Polarity therapy  1x 60-75 min on Tuesdays for one week.  Arm B:  N=5  Polarity therapy  2x 60-75 min Tuesdays over two weeks  Arm C:  N=5  Control arm, standard care | 1. (Influence of fatigue on) quality of life with FACIT-F | 1. Comparison of arms: at T1: significant improvement in both PT arms (MCS= 3.8, SD= 13.5) compared to control arm (MCS= -8.0, SD= 5.6), p= 0.02; at T2: no significant differences between the arms, p= 0.68 |
| Samarel (1998) | Randomized controlled study | n=35  Breast cancer (no information about stage)  Attrition=4 (3x OP cancellation, 1x inability to make second home visit)  Drop-Out: N/A  Evaluated: n=31 | Arm A:  N=14  Preoperative and postoperative  10 min Therapeutic Touch (TT) and 20 min dialogue  Arm B:  N=17  Control arm, preoperative and postoperative  10 min rest and 20 min dialogue | 1. Pain with VAS-Pain | 1. Comparison of both arms: no significant postoperative differences between TT and control arm, p= 0.972 |
| Tabatabaee (2016) | Randomized controlled study | n=90  Various cancer diagnoses (stage II-IV)  Attrition=0  Drop-Out: N/A  Evaluated: n=90 | Arm A:  N=30  Therapeutic Touch  Arm B:  N=30  Placebo  Both arms: 7 applications with an interval of 3 days between each 2 applications for a 4-week period  Arm C:  N=30  Control arm, standard care | 1. Pain interfered with General Activity measured with BPI  2. Pain interfered with Mood measured with BPI  3. Pain interfered with Walking ability measured with BPI  4. Pain interfered with Relation with other people measured with BPI  5. Pain interfered with Sleep measured with BPI | 1. Comparison of arms: at T2: significant differences between TT, placebo and control arm, p< 0.001; no significant differences between placebo and control arm, p= N/A  2. Comparison of arms: at T2: significant differences between TT, placebo and control arm, p< 0.001; no significant differences between placebo and control arm, p= N/A  3. Comparison of arms: at T2: significant differences between TT, placebo and control arm, p< 0.001; no significant differences between placebo and control arm, p= N/A  4. Comparison of arms: at T2: significant differences between TT, placebo and control arm, p< 0.001; no significant differences between placebo and control arm, p= N/A  5. Comparison of arms: at T2: significant differences between TT, placebo and control arm, p< 0.001; no significant differences between placebo and control arm, p= N/A |
| Tsang (2007) | Randomized controlled study | n=16  Various forms of cancer (62.5% colorectal cancer, 12.5% each breast cancer, stomach cancer and lung cancer), cancer stages I-IV  Attrition=2 (Arm A: 2x, 1x relocation, 1x surgery complications)  Drop-Out =1 (Arm A: 1x, no Baseline Information)  Evaluated: n=14 (probably) | Arm A:  N=8  Reiki  Duration: 45 min (average)  Arm B:  N=8  Time to rest  Duration: 45 min (average)  Arm A: Daily single application for a 5-day period, then no application for a 1-week period (wash-out phase 1), then daily single application for a 2-day period, then no applications for a 1-2 week period (wash-out phase 2), then crossover and conduct regimen Arm B  Arm B:  Daily single application over a 5-day period, then no applications over a 1-week period (Wash-Out Phase), after another 2 weeks crossover and implementation of Scheme Arm A | 1. Quality of life with FACT-G  2. Pain with ESAS | 1. Comparison of both arms: at T2: significant improvement in Reiki compared to rest arm, p= 0.04  2. Comparison of both arms: at T1: no significant differences between Reiki and rest arm, p= N/A |
| ABS: Affects Balance Scale, BFI: Brief Fatigue Inventory, BPI: Brief Pain Inventory, BSI: Brief Symptom Inventory, CESD/CES-D: Center for Epidemiological Studies Depression Scale, CNS: Central nervous system, CR: Cognitive restructuring, ESAS: Edmonton Symptom Assessment System, FACIT-F: Functional Assessment of Chronic Illness Therapy-Fatigue, FACT: Functional Assessment of Cancer Therapy, FACT-B: Functional Assessment of Cancer Therapy-Breast, FACT-G: Functional Assessment of Cancer Therapy-General, FACT & GOG-Ntx: Functional Assessment of Cancer Therapy-Gynecologic Oncology Group-Neurotoxicity, FSI: Fatigue Symptom Inventory, HADS: Hospital Anxiety and Depression Scale, HRQoL: Health Related Quality of Life, HT: Healing Touch, HTCQ: Healing Touch Comfort Questionnaire, MAAS: Mindful Attention Awareness Scale, MCS: Mean change score, PMF: Profile of Mood States–Short Form, POMS: Profile of Mood States, POMS-SF: Profile of Mood States-Short Form, PPTBS: Patient´s Perceptions of Therapy Benefit Survey, PT: Polarity Therapy, RFS: Rhoten Fatigue Scale, RRT: Relaxation Response Therapie, RT: relaxation training, SF-36: Validated questionnaire, SD: Standard deviation, STAI: (Spielberger) State-Trait Anxiety Inventory, TM: Therapeutic Massage, TT: Therapeutic Touch, VAS: Visual analog scale, WHOQoL-100/BREF: World Health Organisation Qualtiy of Life-100/Breviate | | | | | |
