# Supplementary material for: Bioenergy therapies as a complementary treatment: a systematic review to evaluate the efficacy of bioenergy therapies in relieving treatment toxicities in patients with cancer
Source: J Cancer Res Clin Oncol. 2022 Sep 27;149(6):2607–19. doi: 10.1007/s00432-022-04362-x (PMC10129966; doi:10.1007/s00432-022-04362-x)
Supplement: Supplementary file 3 — Supplementary file3 (DOCX 23 KB) [file 432_2022_4362_MOESM3_ESM.docx]

Table 2. Excluded reviews and studies

| References | Study Type | Year | Title | Reason for exclusion |
| --- | --- | --- | --- | --- |
| Agdal et al. | SR | 2011 | Energy Healing for Cancer: A Critical Review | All relevant RCTs have already been examined individually |
| Finnegan-John et al. | SR | 2013 | A systematic review of complementary and alternative medicine interventions for the management of cancer-related fatigue | Too few included studies with bioenergy therapies, High overlap with included RCTs |
| Henneghan and Schnyer | SR | 2015 | Biofield therapies for symptom management in palliative and end-of-life care | Risk of Bias has not been investigated |
| Jain and Mills | SR | 2010 | Biofield therapies: helpful or full of hype? A best evidence synthesis | All relevant RCTs have already been examined individually |
| Joyce and Herbison | SR | 2015 | Reiki for depression and anxiety | Too few included studies with cancer patients |
| Mitchell and Berger | SR | 2006 | Cancer-related fatigue: The evidence base for assessment and management | Too few included studies with bioenergy therapies |
| Newby et al. | SR | 2015 | Interventions that may reduce depressive symptoms among prostate cancer patients: a systematic review and meta-analysis | Too few included studies with bioenergy therapies |
| Sood et al. | SR | 2007 | A critical review of complementary therapies for cancer-related fatigue | Too few included studies with bioenergy therapies |
| Tabatabaee et al. | SR | 2016 | Effect of Therapeutic Touch in Patients with Cancer: a Literature Review | Risk of Bias has not been investigated |
| Thrane and Cohen | SR | 2014 | Effect of Reiki therapy on pain and anxiety in adults: an in-depth literature review of randomized trials with effect size calculations | Risk of Bias has not been investigated |
| Vandervaart et al. | SR | 2009 | A systematic review of the therapeutic effects of Reiki | Too few included studies with cancer patients |
| Cook et al. | RCT | 2004 | Healing touch and quality of life in women receiving radiation treatment for cancer: a randomized controlled trial | Fulltext not available |
| Jain et al. | RCT | 2012 | Complementary medicine for fatigue and cortisol variability in breast cancer survivors: a randomized controlled trial | Only a non-relevant intervention group was examined (biofield healing) |
| Pohl et al. | RCT | 2007 | Laying on of hands improves well-being in patients with advanced cancer | Only a non-relevant intervention group was examined (laying on of hands) |
| Smith et al. | RCT | 2003 | Outcomes of touch therapies during bone marrow transplant | No relevant endpoint |
