# Supplementary material for: Bioenergy therapies as a complementary treatment: a systematic review to evaluate the efficacy of bioenergy therapies in relieving treatment toxicities in patients with cancer
Source: J Cancer Res Clin Oncol. 2022 Sep 27;149(6):2607–19. doi: 10.1007/s00432-022-04362-x (PMC10129966; doi:10.1007/s00432-022-04362-x)
Supplement: Supplementary file 4 — Supplementary file4 (DOCX 181 KB) [file 432_2022_4362_MOESM4_ESM.docx]

Table 3. Methodical quality of the included studies

| Appropriate and clearly focused question | Randomized assignment | Adequate concealment method | Blinding of participants and investigators | Comparability of treatment and control group at the beginning | Only group difference is treatment under investigation | Standard, valid and reliable outcomes measurements | Analysis of all patients in the groups | General minimization of bias | Oxford Level of Evidence |  | |  |
| --- | --- | --- | --- | --- | --- | --- | --- | --- | --- | --- | --- | --- |
| + | + | - | - | ? | ? | + | ? | (+) | 2b | | Aghabati (2010) |  |
| + | + | ? | ? | ? | + | + | ? | (+) | 2b | | Alarcão (2016) |  |
| + | + | - | - | + | + | + | + | (+) | 2b | | Beard (2011) |  |
| + | + | ? | ? | ? | + | + | ? | (+) | 2b | | Catlin (2011) |  |
| + | + | - | - | + | + | + | - | (+) | 2b | | Clark (2012) |  |
| + | + | + | + | + | + | + | - | (++) | 1b- | | FitzHenry (2014) |  |
| + | + | + | + | + | + | ? | ? | (+) | 2b | | Frank (2007) |  |
| + | + | - | - | ? | + | + | + | (+) | 2b | | Giasson and Bouchard (1998) |  |
| + | + | - | - | + | + | + | - | (++) | 1b | | Lutgendorf (2010) |  |
| + | + | ? | ? | ? | ? | ? | ? | o | 2b- | | Matourypour (2015) |  |
| + | + | ? | ? | ? | ? | + | ? | o | 2b- | | Matourypour (2016) |  |
| + | ? | ? | ? | ? | ? | ? | ? | o | 2b- | | Vanaki (2016) |  |
| + | + | - | - | - | - | + | - | (+) | 2b | | Mustian (2011) |  |
| + | + | - | - | + | + | + | - | (+) | 2b | | Olson (2003) |  |
| + | ? | - | - | - | - | + | ? | (+) | 2b | | Orsak (2015) |  |
| + | + | ? | ? | + | - | + | - | (++) | 1b | | Post-White (2003) |  |
| + | + | - | - | + | + | + | + | (+) | 2b | | Potter (2007) |  |
| + | ? | - | - | ? | ? | + | ? | (+) | 2b | | Roscoe (2005) |  |
| + | + | ? | ? | ? | ? | + | ? | (+) | 2b | | Samarel (1998) |  |
| + | + | ? | ? | ? | ? | + | ? | (+) | 2b | | Tabatabaee (2016) |  |
| + | ? | - | - | - | - | + | ? | (+) | 2b | | Tsang (2007) |  |
| (++): high quality; (+): acceptable quality; o: unacceptable | | | | | | | | | | |  | |

Xxx Yes Xxx Can`t say Xxx No
