# Supplementary material for: Bioenergy therapies as a complementary treatment: a systematic review to evaluate the efficacy of bioenergy therapies in relieving treatment toxicities in patients with cancer
Source: J Cancer Res Clin Oncol. 2022 Sep 27;149(6):2607–19. doi: 10.1007/s00432-022-04362-x (PMC10129966; doi:10.1007/s00432-022-04362-x)
Supplement: Supplementary file 5 — Supplementary file5 (DOCX 31 KB) [file 432_2022_4362_MOESM5_ESM.docx]

Table 4. Table risk of bias

| Reference | Study type | Standardized rating of risk of bias | Additional comments on methodology | Evidence Level (Oxford) |
| --- | --- | --- | --- | --- |
| Aghabati (2010) | RCT | SIGN  Positive: 3  Uncertain: 3  Negative: 2  Overall quality: Acceptable | PRO: Validated questionnaires; effort was made to maintain consistent  facilities and environmental conditions  CONTRA: Missing blinding or insufficient description; no baseline control; no consideration of multiple testing | 2b |
| Alarcão (2016) | RCT | SIGN  Positive: 4  Uncertain: 4  Negative: 0  Overall quality: Acceptable | PRO: Ethics vote; Adequate use of statistical analyses and methods; Testing for normal contribution; Detailed description of the statistical analysis; Inclusion of the influence of demographic variables on the result (QoL)  CONTRA: Blinding process questionable; No detailed demographic information; Undetailed timeline; No means or SD (or any numbers) describing the results, besides p-values; Overall no information about size of the effect possible; High correlation between the domains, so results are not independent of each other | 2b |
| Beard (2011) | RCT | SIGN  Positive: 6  Uncertain: 0  Negative: 2  Overall quality: Acceptable | PRO: Ethics vote; Comparability of the groups given; High patient adherence; Appropriate use of statistical analysis and methods; Intention-To-Treat Analysis; Bonferroni Correction for multiple testing  CONTRA: Small sample size; Different intensity of interventions; Brief presentation of the results with only a few statistical numbers; No other intervention with “touch” involved; No Poweranalyis; No testing for normal contribution of the data; Timepoints are not well defined; Unequal group sizes for comparison of anxious/depressed vs. normal participants (subgroup analysis); Participants were predominantly well-educated white men; No information on side effects | 2b |
| Catlin (2011) | RCT | SIGN  Positive: 4  Uncertain: 4  Negative: 0  Overall quality: Acceptable | PRO: Ethics vote; Validated questionnaires and information from Cronbach Alpha; Checking the validity and reliability of the interventions; Comparability of groups for age, round of chemotherapy, frequency of prior experience with CAM and origin; Double blinding; Power analysis  CONTRA: Quasi-randomization; Statistical tables questionable: Different evaluated sample numbers from N=58 bis N=66; Comparability of groups questionable; Incorrect blinding information; Only measured short-term effects, which could also be attributed to attention; No description of how to extract the subscales (secondary endpoints) from survey of overall wellbeing and comfort; No indication of mean values (SD) or other values for the questionnaires; No indication of effect sizes or confidence intervals; Indication of demographic data inaccurate and inadequate; No recording which previous experiences were made with CAM or no inclusion of these in the analysis | 2b |
| Clark (2012) | RCT | SIGN  Positive: 5  Uncertain: 0  Negative: 3  Overall quality: Acceptable | PRO: Ethics vote; Exact study flow chart; Validated questionnaires and specification of the Cronbach’s Alpha; Insight into patient compliance; Detailed and clear presentation of the results; Comparability of groups for demographic and medical characteristics  CONTRA: Different intensities of the interventions; High drop-out; Not enough power for producing statistically significant outcomes; Small sample size; Missing values were replaced by the series mean for the variable; Not clear which patient have taken drugs for the neuropathy during the study; No recording which previous experiences were made with CAM or no inclusion of these in the analysis | 2b |
| FitzHenry (2014) | RCT | SIGN  Positive: 7  Uncertain: 0  Negative: 1  Overall quality: High | PRO: Ethics vote; Therapists were replaced by others during the study to avoid over-focusing the patient on one therapist; Comparability of groups for all important demographic data and clinical variables; Successful verification of blinding after the end of the study; Validated questionnaires and details of the Cronbach Alpha  CONTRA: No standardization of sham intervention; Insufficient power; No intention-to-treat analysis; More women in the control group were working full- or part-time; Participants in the HT group had to change the therapist more often than the sham group; No information about the total number of treatments in the respective groups at the end of the study; No recording which previous experiences were made with CAM or no inclusion of these in the analysis | 1b- |
| Frank (2007) | RCT | SIGN  Positive: 6  Uncertain: 2  Negative: 0  Overall quality: Acceptable | PRO: Ethics vote; Comparability of groups for demographic data, pretreatment characteristics and VAS findings; Prior training of placebo therapists to ensure consistent placebo therapy; Blinding of the patients, the clinical and planning staff of the breast center and the study coordinators; Control of variables through multivariate model  CONTRA: Small sample size; No collection of data from patients who complained about their assigned group; Only measured short-term effects; Inaccurate description of the data collection methods; General only superficial description of the framework; No recording which previous experiences were made with CAM or no inclusion of these in the analysis | 2b |
| Giasson and Bouchard (1998) | RCT | SIGN  Positive: 5  Uncertain: 1  Negative: 2  Overall quality: Acceptable | PRO: Indication of Cronbach's alpha and reliabilities  CONTRA: The author is also a performing therapist; no blinding; no information on effect sizes and power | 2b |
| Lutgendorf (2010) | RCT | SIGN  Positive: 5  Uncertain: 0  Negative: 3  Overall quality: High | PRO: Ethics vote; Comparability of the groups; Various and reliable questionnaires; Age and stage of illness as well as health behavior as permanent covariates for the relevant endpoints; Measurement of participants' expectations in advance; Measurement of long-term effects; Testing for normal distribution of data  CONTRA: After five patients, the study design was changed and a new randomization scheme was applied; Some of the Healing Touch treatments were performed by one practitioner, others by two practitioners (63.5% of the sessions); Different treatment duration and different average number of sessions in the groups; Small sample size; No intention-to-treat analysis | 1b |
| Matourypour (2015)  Matourypour (2016)  Vanaki (2016) | RCT  RCT  RCT | SIGN  Positive: 2  Uncertain: 5  Negative: 0  Overall quality: Low  SIGN  Positive: 3  Uncertain: 4  Negative: 0  Overall quality: Low  SIGN  Positive: 1  Uncertain: 6  Negative: 0  Overall quality: Low | PRO: Ethics vote; Sample size determined by Altman’s Nomogram; Validated and reliable questionnaire; Comparability of groups with statistical values for education, patient's occupation, income, spouse's income, age range; Testing for normal distribution of data; Detailed description of the Therapeutic Touch process  CONTRA: Blinding questionable; Selective reporting, which only makes sense in the combination of the two or three studies; Blinding questionable; Comparability of the groups questionable; Interventions carried out by the researcher; Only short-term effects measured; No record and inclusion of previous experience with CAM; No precise indication of cancer stage or prior treatments, time since diagnosis, comorbidities; Mean intervention duration longer for patients with gastrointestinal disease in history (20-25 minutes) and need for reintervention in 69.4% - but no subgroup analysis or information on how many patients had gastrointestinal disease in history; More high-income patients in the intervention group (16.7%) than in the placebo (5.6%) and control groups (2.8%); No presentation of statistical results of endpoint 3 in the results section - p-value is mentioned in the abstract - no information concerning the analysis, presentation of results makes no sense as only percentages are given; No information whether 1st round CTX or further round; Reporting of mean ranks (endpoint 2) not reliable, as it is not possible to trace back what was selected in the mean or median; No detailed information on questionnaires for primary outcomes and non-transparent presentation of these  PRO: Ethics vote; sample size determined by Altman’s Nomogram; validated and reliable questionnaire; comparability of groups with statistical values for education, patient's occupation, income, spouse's income, age range; testing for normal distribution of data; detailed description of the Therapeutic Touch process  CONTRA: Blinding questionable; Selective reporting, which only makes sense in the combination of the two or three studies; Blinding questionable; Comparability of the groups questionable; Interventions carried out by the researcher; Only short-term effects measured; No record and inclusion of previous experience with CAM; No precise indication of cancer stage or prior treatments, time since diagnosis, comorbidities; Mean intervention duration longer for patients with gastrointestinal disease in history (20-25 minutes) and need for reintervention in 69.4% - but no subgroup analysis or information on how many patients had gastrointestinal disease in history; More high-income patients in the intervention group (16.7%) than in the placebo (5.6%) and control groups (2.8%); No presentation of data from the checklist that was collected before the intervention; Unclear comparability of the groups regarding frequency of vomiting; Unclear if CTX is started at the study time (i.e. 1st round for patients) or whether the patients are in a further round  PRO: Ethics vote; sample size determined by Altman’s Nomogram; validated and reliable questionnaire; comparability of groups with statistical values for education, patient's occupation, income, spouse's income, age range; testing for normal distribution of data; detailed description of the Therapeutic Touch process  CONTRA: No information on the type of randomization and blinding; Selective reporting, which only makes sense in the combination of the two or three studies; Blinding questionable; Comparability of the groups questionable; Interventions carried out by the researcher; Only short-term effects measured; No record and inclusion of previous experience with CAM; No precise indication of cancer stage or prior treatments, time since diagnosis, comorbidities; Mean intervention duration longer for patients with gastrointestinal disease in history (20-25 minutes) and need for reintervention in 69.4% - but no subgroup analysis or information on how many patients had gastrointestinal disease in history; More high-income patients in the intervention group (16.7%) than in the placebo (5.6%) and control groups (2.8%); No statistical table for intensity of nausea; No comparison and p-value for placebo and control group (or at least values not provided); VAS (nausea intensity) results before intervention/CTX but not after; Unclear measurement of nausea intensity after intervention; Unclear results of the VAS; No values for individual time points (morning, noon, evening, night); Generally superficial or even selective reporting that questions the interpretation of the results | 2b- |
| Mustian (2011) | RCT | SIGN  Positive: 3  Uncertain: 0  Negative: 5  Overall quality: Acceptable | PRO: Ethics vote; Comparability of groups in terms of demographic variables; Detailed description of massage technique; Baseline differences included as a control variable in analyses; Standardized daily and time intervals for interventions and data collection; Comprehensible statistical analyses; Critical review of own approach and results; Massage therapists with experience with cancer patients; Thorough statistical control of analyses and implementation of corrections (Bayesian analysis as a control for small samples and high SD); Intention to minimize dialogues between the practitioner and the patient  CONTRA: Small sample size; At T0: higher fatigue scores in arm A (M = 3.7, SD = 2.3) and arm B (M = 3.0, SD = 1.7) compared to arm C (M = 1.8, SD = 1.4, p = 0.03) in BFI as well as in diaries (p = 0.003); No standardization of additional treatments; Inconsistent information in tables and text; Bias in subjective endpoints possible, as participants were informed of the aim of the study; No intention-to-treat analysis; Drop-out insufficiently described; No standardization of participant medications; No record and inclusion of experience with CAM; No comparison and p-value on secondary measurement points between intervention and control arms | 2b |
| Olson (2003) | RCT | SIGN  Positive: 5  Uncertain: 0  Negative: 3  Overall quality: Acceptable | PRO: Computer-generated random number assignment system; group assignment was strictly maintained  CONTRA: No blinding or placebo group; just looking at differences between changes; hardly any information on effect sizes and power | 2b |
| Orsak (2015) | RCT | SIGN  Positive: 2  Uncertain: 2  Negative: 4  Overall quality: Acceptable | PRO: Ethics vote; Group comparability for all key demographics, cancer stage, and tobacco and alcohol consumption; Validated questionnaires; No influence of environmental factors; Long-term effects were measured in addition to short-term effects; Clear presentation of the hypotheses; Inclusion of age and cancer stage in the analysis; Bonferroni correction for multiple testing  CONTRA: Differences between groups in quality of life and mood at baseline of the study; No information about randomization method; Data for the control group were not collected simultaneously with the other two groups; Training level of therapists was not the same (1x master level vs. 5x level II); Different group sizes and very small samples; No record and inclusion of experience with CAM | 2b |
| Post-White (2003) | RCT | SIGN  Positive: 4  Uncertain: 2  Negative: 2  Overall quality: High | PRO: Detailed and adequate analysis; power analysis  CONTRA: Hardly any information about blinding; patients left the study because they were not in the desired group | 1b |
| Potter (2007) | RCT | SIGN  Positive: 6  Uncertain: 0  Negative: 2  Overall quality: Acceptable | PRO: Ethics vote; Comparable groups (although p-values were not reported); Scheduled supervisory meetings to review the study protocol, documentation, and therapeutic concerns; Validated questionnaires and data of Cronbach's alpha; Power analysis; Verification of validity and reliability of interventions  CONTRA: Small sample size with only 20% predictive power + Anxiety and depression scores were already low at the beginning of the study (floor effect); Possible selection bias (patients work in university setting, many health professions); No p-values for differences between Reiki and control group; No information on actual measurement times = superficial presentation of results with little statistical evidence; No indication of confidence intervals or effect sizes; No record and inclusion of experiences with CAM; Only data collection at T2 for STAI; No placebo arm | 2b |
| Roscoe (2005) | RCT | SIGN  Positive: 2  Uncertain: 4  Negative: 2  Overall quality: Acceptable | PRO: Ethics vote; Validated and reliable questionnaires; Minimization of conversations between therapist and patient; Clear and comprehensible presentation and collection of data  CONTRA: Demographic and medical data insufficiently described; Very small and homogeneous study population; No placebo or comparison arm; Completion of questionnaires may not always have occurred at the same time (patient-dependent period Tuesday after intervention until Friday evening, submission on Monday) -> Potential source of interference with other influencing factors on the day of data collection by patients; No record and inclusion of experiences with CAM; Unclear randomization process; Number of people in intervention group and feedback reporting does not add up (11 out of 10) | 2b |
| Samarel (1998) | RCT | SIGN  Positive: 3  Uncertain: 5  Negative: 0  Overall quality: Acceptable | PRO: Standardized treatment protocols; Reliable randomization; Validated questionnaires; Groups comparable in terms of demographic data and cancer treatment  CONTRA: No information regarding ethics vote; Small sample size (original study design with 60 participants); Different group size (n=14 vs. n=17); Large preoperative and postoperative range (1-7 days) in which treatments are performed; No baseline measurement + No testing for differences in relevant study variables between groups prior to treatment initiation; Pre-selection of study participants by surgeons; Information on demographic data inaccurate and insufficient; No record and inclusion regarding CAM experience; No exact information about possible effect of Co-intervention (piano music, dialogue, staying at home); Double blinding questionable - hard to believe that no nurses or patients in the hospital talked about the study design; Superficial presentation of results | 2b |
| Tabatabaee (2016) | RCT | SIGN  Positive: 3  Uncertain: 5  Negative: 0  Overall quality: Acceptable | PRO: Ethics vote; Comparability of groups by age, education, chronic diseases, cancer stage; Validated and reliable questionnaires; Listing of means and standard deviation (SD) for questionnaires; Bonferroni correction for multiple testing; Appropriate use of statistical analyses and methods  CONTRA: No standardization of intervention in the placebo group; No p-values for comparison of arm B and C; No indication of effect sizes or confidence intervals; No record of previous experience with CAM; No information on cancer diagnosis, previous treatment (RTX, CTX, OP) and remission date; No information about chronic diseases; No information how physicians recorded cancer-related pain, how pain intensity was measured and whether it was comparable between study groups at baseline; Unclear baseline comparison of variables that affect endpoints; Timeline not clearly described; no detailed description of procedure and patients; No description of the BPI scale; No information on blinding | 2b |
| Tsang (2007) | RCT | SIGN  Positive: 2  Uncertain: 2  Negative: 4  Overall quality: Acceptable | PRO: Ethics vote; Validated and reliable questionnaires; Exact description of the study procedure  CONTRA: Important demographic and medical data were not collected; No exact statement how many patients were evaluated; Different duration of Reiki treatments; Different duration of the second wash-out phase; Lower number of participants than originally planned (instead of 20 only 16 participants); Recommendation of Reiki therapy by patients' oncologists -> Only patients convinced of the therapy participated; No placebo or active control group; No monitoring and validation of rest periods in arm B possible; Different baseline values; No consideration of external influences that could have disturbed "rest" in the control condition (life events, medical events); No record of previous experiences with CAM; Unclear randomization process; 6th and 7th Reiki application was dependent on participants | 2b |
| AMSTAR: A Measurement Tool to Assess Systematic Reviews, RCT: Randomized control trial, SIGN: Scottish Intercollegiate Guidelines Network Methodology: Checklist 2: Randomized Controlled Trials, SR: Systematic Review | | | | |
